# Practices and complications of pubic hair removal among Saudi women

**DOI:** 10.1186/s12905-018-0661-6

**Published:** 2018-10-22

**Authors:** Abdulrahim A. Rouzi, Rigmor C. Berg, Jamela Turkistani, Rana Alamoudi, Nawal Alsinani, Souzan Alkafy, Ahmad Alwazzan

**Affiliations:** 1 Department of Obstetrics and Gynecology, King Abdulaziz University, PO Box 80215, Jeddah, 21589 Saudi Arabia; 2 Norwegian Institute of Public Health, Oslo, Norway; 3 Department of Community Medicine, The University of Tromso, Tromso, Norway

**Keywords:** Pubic hair, Removal, Practices, Complications

## Abstract

**Background:**

Pubic hair grooming, including the complete removal of pubic hair, has become an increasingly common practice, particularly among young women. Although widespread, there is limited data regarding the methods, products, reasons, and complications of pubic hair removal, particularly among Saudi women. The objective was to examine pubic hair removal practices and the prevalence of its complications among Saudi women living in Jeddah, Saudi Arabia.

**Methods:**

In this cross-sectional study conducted at King Abdulaziz University Hospital, Jeddah, Saudi Arabia, Saudi women between 16 and 60 years of age who had the ability to read and speak Arabic, were eligible to complete an anonymous and self-administered survey on pubic hair removal practices and its complications.

**Results:**

Between December 2015 and September 2016, 400 Saudi women completed the survey. The age was 26.3 ± 6.9, 16–58 (mean ± SD, range) years. About three quarters (77.0%) self-removed their pubic hair, while the remainder made use of professional personnel in medical clinics (15.5%), beauty salons (5.3%), and professional services at home (2.2%). Many women (41.8%) used a combination of hair removal methods, with non-electric razor as the most common single method used (33.5%), followed by laser (8.7%), sugaring (6.0%), waxing (4.5%), trimming (2.0%), electric razor (2.0%), and cream (1.5%). Three-quarters of women (75.5%) reported complications, and although they were mostly minor injuries, treatment had to be sought for 17.9% of complications. Multivariable analyses showed that no variables remained correlated with the occurrence of complications (age of starting hair removal, income, BMI, level of education, mode of removal, advice on removal).

**Conclusions:**

Saudi women initiate pubic hair removal in early adolescence. While most complications are minor, close to one in five women experience complications.

## Background

Throughout the ages, humans have modified their body and head hair for functional and aesthetic reasons. Pubic hair removal is a more recent, but increasingly common grooming practice and shows a great range of variability between different populations [1–6]. Although carried out by both males and females, the practice is seen more frequently in women. In one study performed at a large Midwestern University in the United States, 95% of the male and female participants had removed their pubic hair in the previous 4 weeks [5]. Total pubic hair removal is becoming more prominent in society at large. However, research suggests that the majority of women usually leave some hair in their genital area. In a study of 2451 women aged between 18 and 68 years, complete pubic hair removal was particularly more prevalent among young women [7]. Complete pubic hair removal was also correlated with higher female sexual function index scores and more positive genital self-image according to the Female Genital Self-Image Scale [7].

Some demographic differences in pubic hair grooming have been reported [8–11]. For example, in a study performed on a cross-section of women in the U.S. (*n* = 3316), pubic hair grooming was reported to be most strongly associated with being white, younger, and educated [9]. Other studies from the U.S. have identified being of under- or normal weight, having a greater interest in sex and more lifetime sexual partners as associated with pubic hair grooming [8, 10, 11].

Numerous reasons exist for removing pubic hair, including hygiene, comfort, aesthetic reasons, sex appeal (often associated with being partnered), receiving cunnilingus, having looked at one’s genitals in the previous 4 weeks, and some may feel pressured by family or friends to participate in hair removal practices [5–7, 12]. While religion has not yet been examined related to pubic hair removal, in Muslim culture today, both men and women are encouraged to remove armpit and pubic hair [13]. A recent study in Turkey found that the vast majority of Turkish Cypriot women regularly removed their pubic hair [14].

A multi-billion-dollar industry has developed around the numerous methods and products available for hair removal. Products and techniques include shaving (most common method, performed with a razor/electric razor), waxing, threading/plucking, trimming with scissors, depilatory cream, sugaring (use all-natural paste or gel), dyeing/bleaching, electrolysis, and laser [7, 9, 12, 15]. Although considered a safe grooming behaviour, hair removal can result in adverse health events depending on the method used. These complications can include ingrown hairs, epidermal abrasion, folliculitis, vulvitis, or contact dermatitis. In more serious cases, genital burns can occur from waxing, and severe skin irritation from various products can lead to vaginal irritation or post inflammatory hyperpigmentation [16–18]. Disconcertingly, pubic hair modification is also related to self-reported sexually transmitted infection (STI) history [19].

Despite the widespread nature of pubic hair removal, there is little formal research on the practice in general, and among women from diverse religious and ethnic backgrounds in particular. The current study examined pubic hair removal practices and prevalence of its complications among Saudi women living in Jeddah, Saudi Arabia.

## Methods

This observational study was approved by the Ethics Research Committee of King Abdulaziz University Hospital, Jeddah, Saudi Arabia, and performed by relevant guidelines and regulations in Saudi Arabia. Between December 1, 2015, and September 1, 2016, all Saudi women who visited the gynecology clinic at King Abdulaziz University Hospital in Jeddah were invited to participate in the study. The recruitment was done by one of the authors, in private, during the consultation. In addition to being Saudi, eligibility criteria were being between 16 and 60 years old, and able to read and speak Arabic. Selected clinic staff were trained by study team members to recruit eligible and agreeing women, obtain written informed consent, administer the questionnaire in the waiting area, answer any questions, and submit the completed surveys to team members for data entry. The self-completed survey designed for this study included questions on demographics (age, education, religion), the age of pubic hair removal initiation, current removal practices, whether they had received advice on pubic hair removal and from whom, and complications related to pubic hair removal. In total, it included 20 questions and took about 10 min to complete. The BMI was calculated by taking women’s height and weight in the clinic.

The data were analyzed using the Statistical Package for the Social Sciences (SPSS Inc., Chicago, IL, USA), version 23.0. Logistic regression models were used to identify possible predictors (age of starting hair removal, BMI < 25 kg/m^2^ or ≥ 25 kg/m^2^, income < 20,000 Saudi Riyal or ≥ 20,000 Saudi Riyal, level of education ≤high school vs university, mode of removal (self vs other), frequency of removal, and advice on removal (yes/no) of complications. All variables were dichotomous, except age and frequency of removal, which were continuous. *P* < .05 indicated statistical significance.

## Results

During the nine-month recruitment period, a convenience sample of 422 Saudi women were invited to participate. Five percent (*n* = 22) of women declined participation, and 400 women completed the survey. Sample characteristics, current practices, and complications of pubic hair removal are shown in Table 1. The age was 26.3 ± 6.9, 16–58 (mean ± SD, range) years and close to half (47.7%) had a university-level education. They were all Muslims. The weight classification based on BMI was mostly normal (57.0%), but a third (32.8%) of the women were overweight or obese. All women reported removing their pubic hair. The average age of pubic hair removal initiation was 13.5 ± 1.9 years (range, 8–21 years). The frequency of removal was 20.8 ± 14.6 days (range, 3–90 days). The vast majority of women (77.0%) self-removed their pubic hair, while the remainder made use of professional personnel in medical clinics (15.5%), beauty salons (5.3%), and professional services at home (2.2%). The method used for hair removal was primarily a combination of several methods (41.8%) and by using a razor (33.5%), but other methods, such as laser, sugaring, cream, and waxing were also reported. Similarly, reasons for pubic hair removal were diverse, with 65.8% reporting they did it for a range of reasons. Among those who stated there was one reason, this was specified as appearance (18.5%), hygiene (9.0%), and religion (Islam) (5.5%). Two-thirds of women (62.5%) had sought and received advice on hair removal, which most commonly came from the participants’ mother (68.8%). Few (4.0%) stated that the advice came from a physician.

**Table 1:** Sample characteristics, current practices of pubic hair removal, and complications among Saudi women (*n* = 400) in Jeddah, Saudi Arabia

Age, years	26.3 ± 6.9 (range = 16–58)
Religion	
Muslim	400 (100)
Education level	
High school or lower	209 (52.3)
Bachelor’s degree or higher	191 (47.7)
Body mass index	
Underweight (BMI < 18.5)	24 (6.0)
Normal (BMI 18.5–25)	228 (57.0)
Overweight (BMI 25–30)	92 (23.0)
Obese (BMI > 30)	39 (9.8)
Missing	17 (4.2)
Monthly income	
< 5000 Saudi Riyal (<≈1330 US$)	145 (36.3)
5000–10,000 (≈1331–2665 US$)	127 (31.7)
10,000–20,000 (≈2666–5330 US$)	65 (16.3)
> 20,000 (> ≈ 5331 US$)	43 (10.7)
Missing	20 (5)
Age of pubic hair removal initiation	13.5 ± 1.9 (range = 8–21)
Frequency of hair removal (days)	20.8 ± 14.6 (3–90)
Mode of removal	
Self	308 (77.0)
Medical clinic	62 (15.5)
Beauty salon	21 (5.3)
Home service	9 (2.2)
Method of removal	
Razor blade	134 (33.5)
Laser	35 (8.7)
Sugar	24 (6.0)
Wax	18 (4.5)
Electric razor	8 (2.0)
Trim with scissors	8 (2.0)
Cream	6 (1.5)
Pluck	0
Combination of methods	167 (41.8)
Reason for removal	
Appearance	74 (18.5)
Hygiene	36 (9)
Religion	22 (5.5)
Combination of reasons	263 (65.8)
Missing	5 (1.2)
Has sought and received advice on hair removal	
No	150 (37.5)
Yes	250 (62.5)
Has received advice on hair removal from	
Mother	172 (68.8)
Sister	39 (15.6)
Friend	17 (6.8)
Physician	10 (4.0)
Self-reading	12 (4.8)
Clinical complications	
Yes	302 (75.5)
No	98 (24.5)
Type of complication	
Cuts	31 (10.3)
Severe itching	30 (9.9)
Ingrown hairs	27 (8.9)
Rash	12 (4.0)
Burn	10 (3.3)
Allergy	8 (2.6)
Bruise	6 (2.0)
Abrasion	6 (2.0)
Hyperpigmentation	6 (2.0)
Combined	166 (55.0)
Received treatment for complication	
Yes	54 (17.9)
No	244 (80.8)
Missing	4 (1.3)

Data are mean ± SD (range) or number (percentage)

As seen in Table 1, three-quarters of the respondents (75.5%) self-reported they had experienced complications from pubic hair removal. Specifically, complications included skin cuts (10.3%), severe itching (9.9%), ingrown hair (8.9%), rash (4%), burn (3.3%), allergy (2.6%), bruises (2%), abrasions (2%), hyperpigmentation (2%), or a combination of complications (55%). About one in five women (17.9%) required treatment for their pubic hair removal complication. Multivariable analyses showed that none of the variables remained correlated with the occurrence of complications (age of starting hair removal, BMI, the level of education, mode of removal, income, frequency of removal, and advice on removal) (Table 2).

**Table 2:** Multivariable analyses on factors associated with complications

Variable	OR (95% CI)	*P*	AOR (95% CI)	*P*
Age of starting removal	0.89 (0.78–1.04)	0.14	0.93 (0.79–1.10)	0.39
Mode of removal (self or other)	1.04 (0.61–1.80)	0.88	1.00 (0.46–2.20)	1.00
Advice (yes/no)	1.51 (0.95–2.39)	0.08	0.69 (0.38–2.39)	0.32
BMI < 25 and ≥ 25	0.75 (0.46–1.22)	0.25	0.56 (0.28–1.10)	0.09
Income < 20,000 and > 20,000	0.49 (0.25–0.96)	0.03*	0.90 (0.38–2.39)	0.83
Frequency of removal	1.01 (0.99–1.03)	0.44	1.01 (1.00–1.04)	0.28
Education	0.99 (0.63–1.56)	0.96	1.21 (0.63–2.36)	0.56

*CI* confidence interval; all variables are dichotomous except age and frequency of removal, which are continuous

## Discussion

This study examined pubic hair removal practices and associated complications in Saudi women. It adds to the limited data available on pubic hair removal, despite its widespread practice. While there are some research reports in the literature, the majority of these focus on Caucasian, college-aged women from the U.S [5]. The religious etiquettes of Islam specify that removal of pubic hair should be initiated at menarche, and done at least once every 40 days [13, 20]. Accordingly, we found that all respondents removed their pubic hair. Relative to non-Muslim samples, however, we found that pubic hair removal began at an earlier age in this Saudi population (average age 13.5). For example, among 1677 women in the Texas Gulf Coast region, Demaria and Berenson found that the age of initiation for pubic hair removal was 18.35 ± 4.34 years (Mean ± SD) for Hispanic women, 17.52 ± 3.68 years for Black, and 16.40 ± 3.87 years for White women [10].

Interestingly, religion (Islam) was given as a reason for pubic hair removal among 5.5% of participants. Rather, the majority of the participants reported a combination of reasons, including appearance and hygiene. This is similar to the only other known study with a Muslim sample, among Turkish Cypriot women, which found that the main reasons for pubic hair grooming were feeling comfortable and preventing odor [14]. Additionally, also in this study, the vast majority of women preferred traditional methods of pubic hair removal, most commonly waxing. In our study, the most common method was shaving. Also, the preferred source of advice and knowledge on pubic hair removal in both studies were the participant’s mothers: 68.8% in the current study and 70.5% in the Turkish Cypriot study [14]. Muallaaziz and colleagues suggest that this finding indicates that young Turkish Cypriot women are still tied to tradition as they continue to regard the elder women as reliable sources of information despite the availability of other informative sources and trends [14].

We found that complications were commonly experienced (75.5% said they had experienced complications). Although these were primarily minor injuries such as cuts, bruises, itching, some complications did require treatment (17.9%). This is consistent with the literature as minor complications from pubic hair removal are common and have been reported in previous studies [1, 5, 11, 12, 18]. Hair removal injuries can, however, be more serious and require medical attention. A review of the National Electronic Injury Surveillance System (NEISS) in the United States revealed a fivefold increase in emergency department visits due to grooming related genitourinary injuries between 2002 and 2010. The study estimated that there were 11,704 (95%CI 8430–15,004) grooming related genitourinary injuries during this study period [12]. Furthermore, one-third of these injuries were recorded between 2009 and 2010, indicating a significant increase in more recent years [17]. Most of these injuries were due to razor related cuts and lacerations or waxing burns. Molluscum contagiosum, follicular keratosis requiring excision, staphylococcal infections and abscesses are also complications of pubic grooming, especially from waxing [15, 21–25]. In the current study, 5.3% of women attended beauty salons to have their pubic hair removed. Although uncommon, there is also a risk of contracting sexually transmitted diseases from waxing salons, and reports of primary genital herpes from contaminated waxing tools have been described in the literature [26].

Studies on women’s pubic grooming habits contribute to our understanding of the prevalence of removal, methods, and motivations of pubic hair removal. These studies, however, are not without their limitations. The participants of these studies are often self-selected and volunteer to participate because they are interested in the survey topic [27]. Studies conducted on university samples also report on only a small and select segment of the population. In studies conducted in the U.S., Canada, and Australia, the participants are overwhelmingly heterosexual, white females. Therefore, results from these studies cannot be extrapolated to larger racially, sexually or culturally diverse populations [27]. Future studies conducted on more diverse populations will help generate a better understanding of pubic grooming practices. With the increasing number of Muslim women in the West, it is important to be aware of this community’s unique cultural and religious beliefs that affect patient care [28]. Our study comes with strengths and limitations. To the best of our knowledge, this is the first study to examine the pubic hair removal practices and its complications among Saudi women. However, the study was hospital-based, and the sample is non-random.

## Conclusion

Grooming of pubic hair is currently considered a ‘social norm,’ however, very little data on the topic exists. The results of this study are consistent with previous reports in different study populations. One disparity exists, and that is the age of initiation of pubic hair removal. Saudi women appear to begin this practice at an earlier age (~ 13 years) which likely corresponds to menarche. The reported complications, however, are similar, and it is important to note that although serious injuries are uncommon, they do occur. Health advice and emphasis on safe hair removal practices would be beneficial and may help prevent both minor and severe grooming related injuries.
